# Ytterbium-Phosphate Glass for Microstructured Fiber Laser

**DOI:** 10.3390/ma7064723

**Published:** 2014-06-19

**Authors:** Ryszard Stępień, Marcin Franczyk, Dariusz Pysz, Ireneusz Kujawa, Mariusz Klimczak, Ryszard Buczyński

**Affiliations:** 1 Institute of Electronic Materials Technology, 133 Wolczynska Str., 01-919 Warsaw, Poland; E-Mails: ryszard.stepien@itme.edu.pl (R.S.); Marcin.Franczyk@orange.com (M.F.); dariusz.pysz@itme.edu.pl (D.P.); ireneusz.kujawa@itme.edu.pl (I.K.); mariusz.klimczak@itme.edu.pl (M.K.); 2 Department of Physics, University of Warsaw, 7 Pasteura Str., 02-093 Warsaw, Poland

**Keywords:** phosphate glass, ytterbium oxide, glass melting, glass properties measurements, photonic crystal fiber (PCF), fiber laser

## Abstract

In the paper, we report on the development of a synthesis and melting method of phosphate glasses designed for active microstructured fiber manufacturing. Non-doped glass synthesized in a P_2_O_5_-Al_2_O_3_-BaO-ZnO-MgO-Na_2_O oxide system served as the matrix material; meanwhile, the glass was doped with 6 mol% (18 wt%) of Yb_2_O_3_, as fiber core. The glasses were well-fitted in relation to optical (refractive index) and thermal proprieties (thermal expansion coefficient, rheology). The fiber with the Yb^3+^-doped core, with a wide internal photonic microstructure for a laser pump, as well as with a high relative hole size in the photonic outer air-cladding, was produced. The laser built on the basis of this fiber enabled achieving 8.07 W of output power with 20.5% slope efficiency against the launched pump power, in single-mode operation *M*^2^ = 1.59, from a 53 cm-long cavity.

## 1. Introduction

Significant progress has been made over the past few years in the development of laboratory-based and commercial high-power Yb^3+^-doped silica fiber sources [1,2,3,4]. Fused silica is a very useful host for some applications where very long amplifiers can be used. However, it is generally found to be less suitable for small laser devices, because the rare earth concentration is limited by ion clustering, low solubility, concentration-quenching [5], photodarkening [6] and other undesirable effects [7]. In silica, the Yb_2_O_3_ concentration can only be increased maximally to the level of 1–2 wt%. More complex silicate glasses can overcome some of these problems. However, the Yb^3+^ emission cross-section and the laser efficiency is generally low for these glasses. Phosphate glasses allow one to overcome most of these problems [7]. These hosts are attractive laser oscillator/amplifier materials, because unlike silicate, fluoride and other laser glass materials, they combine such useful properties, as good chemical and mechanical durability, ion-exchangeability, high cross-sections for stimulated emission, high gain, low concentration quenching and no clustering effects, low upconversion losses, a wide bandwidth capability, and they can be readily drawn into fibers of complex design. Phosphate glasses also exhibit a very high solubility for rare earth ions and high photodarkening resistance. These features permit the introduction of large concentrations of active ions into relatively small volumes, resulting in smaller laser devices with high energy storage capabilities. The high concentration of rare earth ions in phosphate glasses has several advantages. These high doping concentrations result in highly efficient pumping in short lengths of fibers, high energy storage per unit volume, high gain per unit length and very rapid and efficient energy transfer between rare earth ions. In comparison with the standard silica fiber laser, these properties translate into opportunities for new robust and high power designs.

Photonic crystal fiber (PCF) lasers are the subject of intense evolution. Their properties brought them to an important position in fiber laser applications. In the case of single frequency operation, PCF laser fibers with a double-clad structure are more efficient than step-index fibers if the air-cladding is implemented. For a silica PCF laser, the fiber length can be limited to a few meters or parts of a meter [8], but a step-index laser fiber usually needs to be tens of meters long to provide similar high power operation [9]. Additionally, due to the natural ease of the scaling of PCF structures, large mode area (LMA) fiber laser manufacturing is possible [10]. In order to provide high power solutions, the use of PCF seems to be more promising than the case of step-index fiber lasers [11].

Due to the higher level of rare ion solubility in phosphate glasses than in silica, the length of the laser phosphate fiber can be substantially decreased, even below ten centimeters, at watt-level single-frequency operation [12]. Although phosphate glass fiber suffers from higher propagation loss than silica fiber, there is the substantial prospect for phosphate glass PCF in a laser application in a compact arrangement [13].

In this paper, we report on the development of a thermally stable Yb_2_O_3_-high-doped phosphate glass synthesized in a P_2_O_5_-Al_2_O_3_-Yb_2_O_3_-BaO-ZnO-MgO-Na_2_O oxide system. The doped glass material was prepared to carefully match the refractive index and thermal properties (expansion coefficient, rheology) to the meta-phosphate undoped glass, which was developed within our earlier research projects. Both glasses are provided for the microstructured laser fiber manufacturing.

## 2. Results

All studied glasses have a medium refractive index *n*_D_ = 1.5280–1.5359, measured for the yellow line of sodium λ = 589.6 nm, which is characteristic for crown-type optical glasses (Table 1). When some glass components are replaced by BaO, ZnO or Yb_2_O_3_, the higher atomic weight of the metals causes an increase in the electronic density and polarizability of the glass with an important effect on the refractive index. The highest refractive index values were measured for IRF-16/6Yb/A-C glasses, containing the largest amount of BaO + ZnO + Yb_2_O_3_ at a level of 17 mol% (Table 2). Barium oxide absence in IRF-16/6Yb/D-E glasses, in which a sum of ZnO and Yb_2_O_3_ was 14 mol% and 12 mol%, respectively, caused the clear decreasing of their refractive index to the value of *n*_D_ = 1.5280. IRF-16/6Yb/F glass has *n*_D_ intentionally very close to a value measured for IRF-16/02 Yb_2_O_3_-undoped cladding glass.

**Table 1:** Basic properties of the IRF-16/6Yb/A-F series glasses and IRF-16/02 glass.

Property		IRF-16/Yb/						IRF-16/02
		A	B	C	D	E	F	
Refractive index *n*_d_		1.5359	1.5338	1.5341	1.5280	1.5280	1.5296	1.5305
Transmittance at λ = 750 nm for 2 mm thickness (T decreased due to Fresnel refractive losses) (%)		91.9	91.5	91.8	92.4	92.2	92.1	92.1
Transmittance at λ = 1.55 μm for 2 mm thickness (T decreased due to Fresnel refractive losses) (%)		91.4	91.3	91.2	91.7	91.4	91.2	91.3
Transmittance at λ = 2.5 μm for 2 mm thickness (T decreased due to Fresnel refractive losses) (%)		86.6	85.1	84.7	83.1	79.0	81.0	81.4
Short-wave absorption threshold λ_S_ (nm)		239–241						
Absorption coefficient for λ = 975 nm (cm^−1^)		16.06	17.18	17.48	18.06	18.81	18.53	0.0046
Thermal expansion coefficient for the range of 20–300 °C α (10^−7^·K^−1^)		85.0	90.0	90.0	89.3	89.7	92.3	91.1
Thermal expansion coefficient for the range of 20–400 °C α (10^−7^·K^−1^)		88.0	95.0	93.0	93.8	93.5	96.3	96.0
Lower annealing temperature *t*_l_ (°C) logη = 14.6		400.0	394.0	395.7	382.3	389.3	378.3	429.5
Transition temperature *T*_g_ (°C) logη = 13.4		479.5	453.8	474.9	462.8	467.7	458.2	482.1
Upper annealing temperature *t*_u_ (°C) logη = 13		492.5	463.6	488.1	475	479.8	472.6	503.8
Dilatometric softening point *T*_d_ (°C) logη = 11.0		517.4	484.1	512.1	496.2	501.2	493.2	529.0
Characteristic temperatures in Leitz heating microscope *T* (°C); temperature of:								
curvature*T*_c_	logη = 9.0	575	520	560	520	520	530	560
sphere*T*_sph_	logη = 6.0	680	630	650	625	640	620	650
hemisphere*T*_hs_	logη = 4.0	740	675	710	670	710	675	685
spreading*T*_spr_	logη = 2.0	850	750	780	760	760	780	830
Crystallization (2 h treatment at *T*_sph_)		Not observed any crystallization traces						
Density ρ (g/cm^3^)		2.95	2.94	2.95	2.89	2.89	2.90	2.74
Vickers Hardness (HV) (GPa)		5.5	5.5	5.5	5.5	5.5	5.5	5.5
Water Durability (WD) (mass decrease after 6 h boiling) (mg/100 cm^2^)		17.9	23.2	20.3	22.1	23.8	26.9	12.6

**Figure 1:**
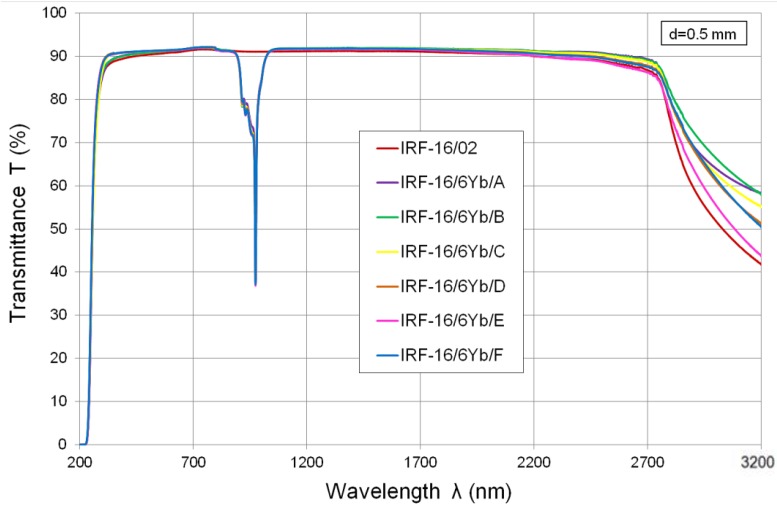
Transmittance of IRF-16/6Yb/A-F series glasses and IRF-16/02 glass from 100-g batch meltings (the spectrum is not corrected for reflection loss).

**Figure 2:**
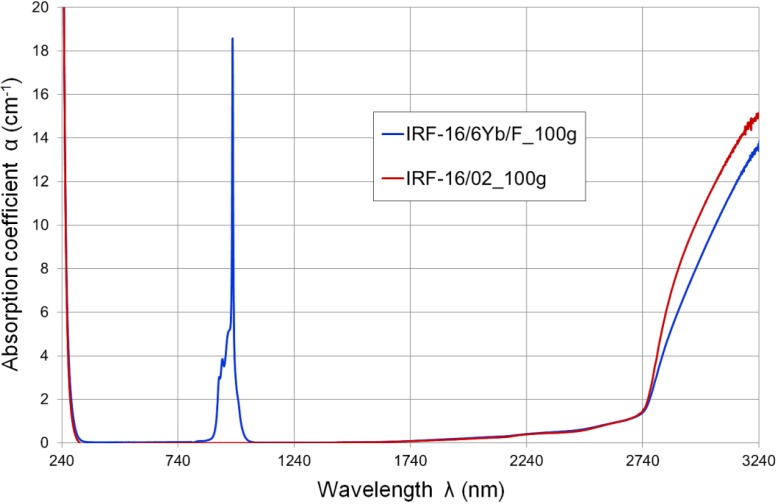
Spectral absorption characteristics of host matrix IRF-16/02 glass and active rod IRF-16/6Yb/F glass (small 100-g batch meltings).

The developed Yb^3+^-doped glasses are characterized by very high transmittance and a low absorption coefficient in a wide spectral range of 320–2700 nm, except for a band of 880–1050 nm with a maximum absorption peak at 975 nm (Figure 1 and Figure 2). Such a spectrum is typical for glasses with ytterbium oxide content. Above a wavelength of 1700 nm transmittance decreases slightly, and above 2740 nm a wide absorption band with a maximum at about 3300 nm can be observed. This is caused mainly by the presence of hydroxyl ions (OH^−^) in glass structure. Selected points of transmission characteristics for developed glasses are summarized in Table 1. Fresnel refractive losses are not considered in presented glass transmission values.

**Table 2:** Transition temperature (*T*_g_), crystallization beginning temperature (*T_x_*) and thermal stability (Δ*T*) of glasses.

Glass Name	*T*_g_ (°C)	*T_x_* (°C)	Δ*T* = *T*_x_ − *T*_g_ (°C)
IRF-16/6Yb/A	479	692	213
IRF-16/6Yb/B	454	657	203
IRF-16/6Yb/C	475	682	207
IRF-16/6Yb/D	463	661	198
IRF-16/6Yb/E	468	687	219
IRF-16/6Yb/F	458	674	216
IRF-16/02	482	703	221

**Figure 3:**
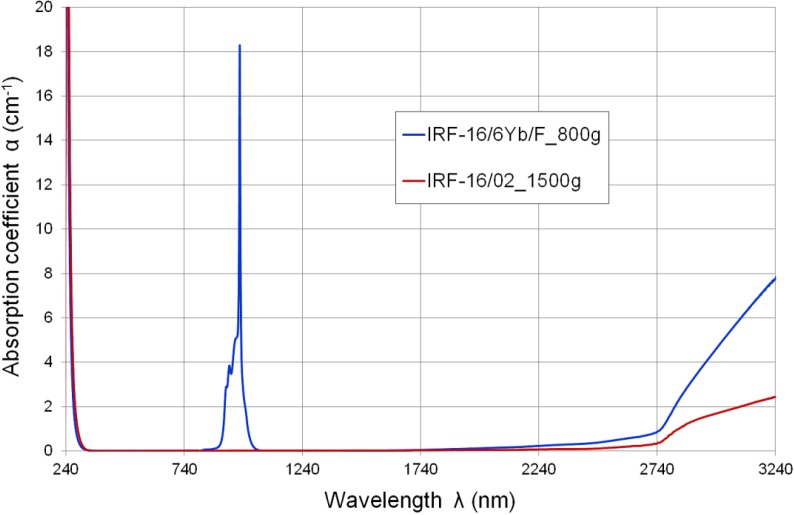
Spectral absorption characteristics of host matrix IRF-16/02 glass and active rod IRF-16/6Yb/F glass (large batch meltings of 1500 g and 800 g, respectively).

The glasses melted in a big volume and with intensive oxygen bubbling applied during the melting process exhibit a much lower OH^−^ ion concentration and, consequently, a much lower absorption coefficient in the infrared region above 1.7 μm, especially at 3.2–3.3 μm (Figure 3).

The coefficient of the thermal expansion (CTE) of glass is the key parameter that influences the thermal shock resistance and is very important when one glass is joined with another in an all-solid glass photonic structure. The CTE of our IRF glasses, equal to 85.0–92.3 × 10^−7^ K^−1^ as determined for the temperature range of 20–300 °C, is very low compared to the other types of phosphate glasses: 100 × 10^−7^ K^−1^ [14] or even 120 × 10^−7^ K^−1^ [15]. Such CTE difference can be explained with the presence of Yb_2_O_3_ and two valent metal oxides, such as MgO and ZnO, which are introduced into our glasses, instead of an excessive amount of alkali oxide, but mainly with the presence of the glass network intermediate, Al_2_O_3_. IRF-16/6Yb/F glass has a CTE closest to the value measured for our base IRF-16/02 glass. The CTE difference is as low as 1.2 × 10^−7^ K^−1^ determined for 20–300 °C or even only 0.3 × 10^−7^ K^−1^ for the 20–400 °C considered temperature range. In terms of CTE, these two glasses are very well matched.

**Figure 4:**
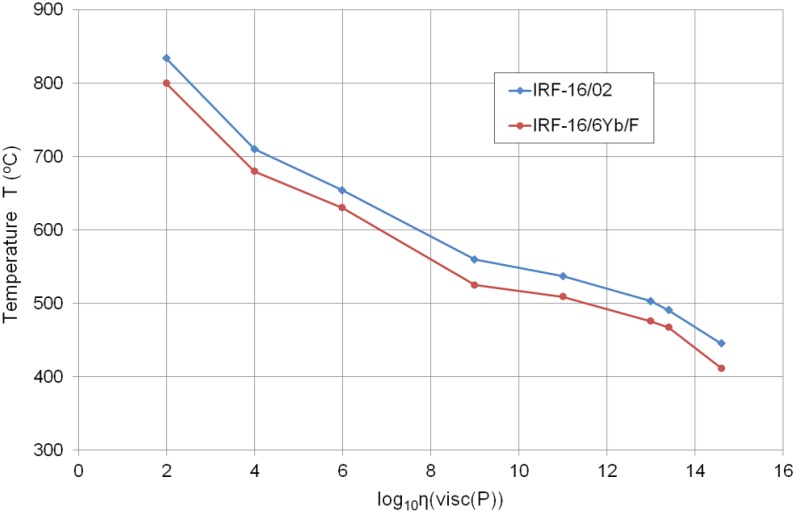
Viscosity characteristics of two finally matched glasses (melting volumes of 1500 g and 800 g).

The measured viscosity characteristics (Figure 4) shows that the developed glasses have a wide processing temperature range, which means that they are technologically long. They can be formed by different methods—from casting up to manual forming and fiber drawing—in a wide glass-working temperature range. If the viscosity range η = 10^4^–10^7.65^ P is considered, the working temperature range (technological length) is above 100 °C for the examined phosphate glasses; hence, it is much wider if compared to, e.g., tellurite [16] or heavy metal lead-bismuth-gallium oxide glasses [17]. The glasses that are characterized by a larger technological length are more suitable for the microstructured optical fiber fabrication technology.

Glasses to be considered suitable for photonic crystal fiber fabrication must possess very good stability against crystallization. A preliminary crystallization test was applied to determine the thermal stability of all developed glasses. After 2 h of thermal treatment at temperature *T*_sph_, determined in a Leitz heat microscope, no crystallites were observed on the sample surfaces when examining under the optical microscope (Table 1). The fact of the high crystallization resistance of fabricated glasses was confirmed through DSC measurements. Only very weak exothermal peaks can be observed on the DSC curves. In Table 2, we show the glass transition temperature (*T*_g_), the onset of crystallization temperature (*T_x_*) and the difference of these temperatures (Δ*T* = *T_x_* − *T*_g_) determined by the DSC method for the synthesized glasses. Usually a temperature difference Δ*T* = *T_x_* − *T*_g_ is a good indicator for glass thermal stability. For almost all of our glasses, this parameter is very high, above 200 °C. Only IRF-16/6Yb/D glass exhibits a lower value equal to 198 °C. The glasses for which Δ*T* is so high are thermally stable, and no crystallization is observed when they are heat treated at softening temperatures (logη = 6–9) for a long term (few hours).

The glasses that offer a high thermal stability (Δ*T* = *T*_x_ − *T*_g_) are the best candidates for PCF fabrication due to their relatively small probability of crystallization problems. The observed great stability is mainly caused by the presence of the network intermediate, Al_2_O_3_. The addition of Al_2_O_3_ to phosphate glasses helps in enhancing the thermal stability against devitrification by increasing the number of bridging oxygen and by forming a more cross-linked chain glass structure [18].

**Figure 5:**
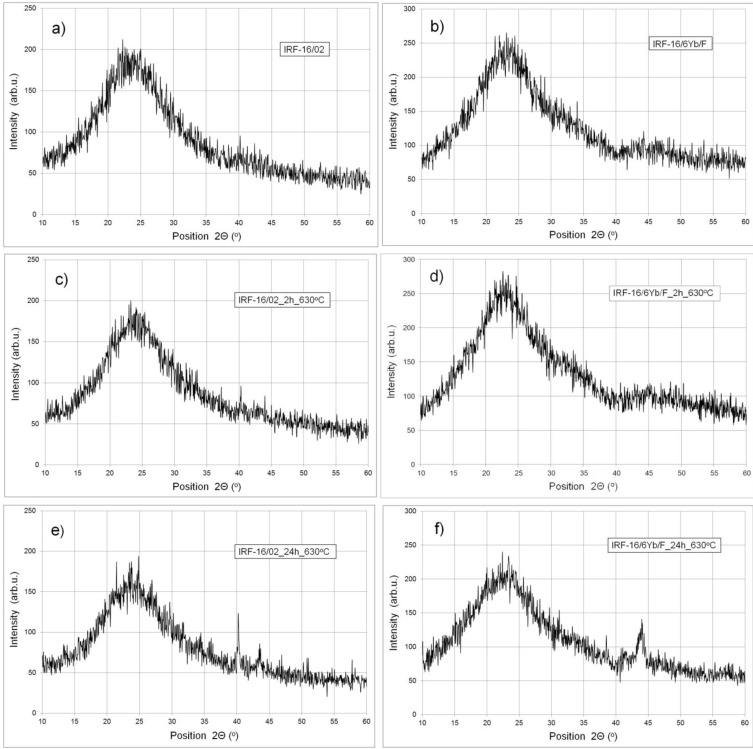
X-ray diffractograms of the IRF-16/02 and IRF-16/6Yb/F glasses (from large batch meltings): (**a**,**b**) without thermal treatment; (**c**,**d**) treated 2 h at 630 °C; (**e**,**f**) after treatment during 24 h at 630 °C.

The XRD measurements confirmed the previously obtained DSC results. The tests were performed on the samples prepared from the 800-g and 1500-g large melts of IRF-16/6Yb/F and IRF-16/02 glasses. Both glasses subjected to 2 h thermal treatment at 630 °C do not show any sign of crystallization. We can observe a high background from the glassy state only (Figure 5). In the case of samples treated 24 h at 630 °C, a few very weak reflections can be observed in the background, but due to their intensity, the determination of the crystalline phases was not possible.

The performed isothermal treatment crystallization test, DSC, as well as XRD measurements confirmed clearly that fabricated glasses are thermally stable, and the photonic fiber drawing process can be accomplished with their participation.

The introduction of Yb_2_O_3_, an oxide with a high molecular weight, caused a density increase of IRF-16/6Yb/A-F series glasses. Their densities, ranging from 2.89 to 2.95 g/cm^3^, are considerably higher if compared to IRF-16/02 glass without Yb_2_O_3_ component (ρ = 2.74 g/cm^3^; Table 1). It is clear that the density changing trend is similar to that one observed for the refractive index.

Microhardness defines the mechanical strength of glasses and determines the durability of the manufactured fibers. Microhardness appears constant for all studied glasses achieving a high value of 5.5 GPa, which is comparable to the value measured for soda-lime silicate glass.

The glasses are characterized by a medium water durability, which ranges from 17.9 to 26.9 mg/100 cm^2^ for Yb_2_O_3_-doped glasses. In the case of undoped IRF-16/02 glass, it is equal to 12.6 mg/100 cm^2^, which is a better result. For a comparison, Schott optical glasses SF57 (lead-silicate) and BK7 (boron-silicate) have a measured water durability as high as 0.4 and 0.1 mg/100 cm^2^ accordingly. However, for the three-component PbO-Bi_2_O_3_-Ga_2_O_3_ glasses, we have measured the water durability to be as low as 180 mg/10 cm^2^ [17]. We must take into account that the higher the value expressed in mg/100 cm^2^, the lower is the water durability. The low water durability of the glass creates considerable difficulties during the grinding and polishing processes of the glass elements in a water environment.

## 3. Glass Synthesis

The thermally stable meta-phosphate glass, labeled here as IRF-16/02 was developed in-house and successfully applied in the fabrication of microstructured fibers [13,19]. Drawn laser fiber structures consisted of a cladding glass joined with an ytterbium-doped phosphate glass rod, in which a concentration of Yb_2_O_3_ was up to 3 mol%. High-power laser applications however require higher dopant concentrations; hence, a new active glass rod had to be fabricated.

Six ytterbium-doped glasses with a Yb_2_O_3_ concentration of 6 mol % (about 18 wt% or 15.7 × 10^20^ Yb^3+^/cm^3^, if the glass density is equal to 2.9 g/cm^3^) were designed. Their compositions result from changing the composition of IRF-16/02 cladding glass synthesized in the P_2_O_5_-Al_2_O_3_-BaO-ZnO-MgO-Na_2_O oxide system (Table 3). Chemical compositions of the IRF-16/6Yb/A-F series rod glasses (Table 4) were chosen by the changing of the concentration of all components, except Yb_2_O_3_, the concentration of which was kept constant, to achieve the high compatibility of the obtained glasses with the base IRF-16/02 cladding glass in the terms of optical, thermo-mechanical and rheological properties, especially their refractive index, thermal expansion coefficient and viscosity characteristics.

**Figure 6:**
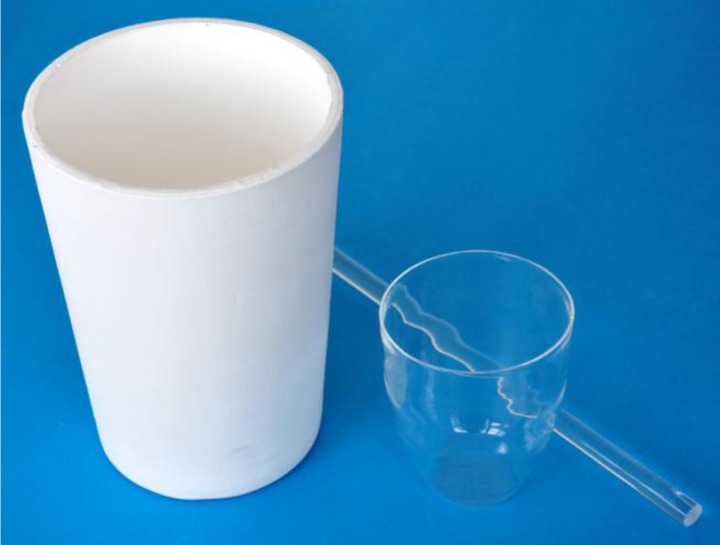
Crucibles applied in the glass melting processes: 1100 cm^3^ in volume made of ground and sintered quartz glass; 200 cm^3^ in volume made of clear quartz glass; 10 mm in diameter quartz glass rod used for glass melt stirring.

The IRF-16/6Yb/A-F series glasses and IRF-16/02 glass were initially prepared following a conventional quenching technique using high purity P_2_O_5_ (Acros Organics pure p.a. >99%), BaCO_3_ (Merck Optipur 99.999%), Ba(NO_3_)_2_ (Alfa Aesar 99.95%), Al_2_O_3_ (Acros Organics extra pure 99.99%), ZnO (Alfa Aesar 99.99%), MgO (Alfa Aesar 99.99%), Na_2_CO_3_ (Chempur Poland pure p.a. >99.7%), NaNO_3_ (Chempur Poland pure p.a. >99.8%), Yb_2_O_3_ (Alfa Aesar REacton 99.99%) and Sb_2_O_3_ (Alfa Aesar Puratronic 99.999%), all in a powder form (except crystalline NaNO_3_). Appropriate amounts of these chemicals were mixed in a porcelain mortar inside a glove box under dried nitrogen atmosphere. The mixed powders (for 100-g portions of glass) were melted in a 200 cm^3^ crucible made of clear quartz glass (Figure 6), within an electrical furnace under air atmosphere. After batch charging at 1150 °C, the temperature was increased up to 1350 °C at a rate of 6 °C/min. The glass melt was mixed two times using silica glass rods (8 mm in diameter, Figure 1). The temperature of the furnace was held at 1350 °C for 1 h, and then, it was decreased to 1220 °C at a rate of 3 °C/min. The glass melt at 1220 °C was cast into a graphite mold preheated to 475 °C. The glass together with the mold was then put into an electric muffle furnace, protected by a nitrogen atmosphere. After holding for 1 h at a temperature of 475 °C, it was cooled down (annealed) slowly to room temperature at a rate of 0.4 °C/min. The glasses were well melted and observed to have no solid, gas or crystalline impurities immediately after casting into the mold, as confirmed by inspection using an optical microscope.

The glasses used for fiber fabrication were melted in portions of 1500 g (IRF-16/02 glass) and 800 g (IRF-16/6Yb/F) in 1100 cm^3^ crucibles made of ground and sintered quartz glass (Figure 6). The glass melt at 1350 °C was intensively bubbled by ultra-clean and dry oxygen (<0.5 ppm of water content). The oxygen bubbling was applied for better homogenization of the glass melt and for a decrease of OH^−^ ions in the glass structure.

**Table 3:** The chemical composition of the IRF-16/6Yb/A-F series glasses compared with the base composition of IRF-16/02 glass (mol%).

Glass Name		Changes in IRF-16/02 Glass Composition
IRF-16/6Yb/	A	3% Al_2_O_3_ + 3% BaO → 6% Yb_2_O_3_
	B	6% Al_2_O_3_ → 6% Yb_2_O_3_ 4% BaO → 1% ZnO + 1% MgO + 2%Na_2_O
	C	4% Al_2_O_3_ + 2% BaO → 6% Yb_2_O_3_ 1% BaO → 1% Na_2_O
	D	6% Al_2_O_3_ → 6% Yb_2_O_3_ 6% BaO → 4% MgO + 2% Na_2_O
	E	6% Al_2_O_3_ → 6% Yb_2_O_3_ 6% BaO → 2% MgO + 2% Na_2_O + 2% P_2_O_5_ 2% ZnO → 2% P_2_O_5_
	F	6% Al_2_O_3_ → 6% Yb_2_O_3_ 5% BaO → 2% MgO + 3% Na_2_O 2% ZnO → 2% P_2_O_5_

**Table 4:** Chemical composition of the IRF-16/6Yb/A-F series glasses and IRF-16/02 glass (mol %).

Glass Name Oxide	IRF-16/6Yb/						IRF-16/02
	A	B	C	D	E	F	
P_2_O_5_	58	58	58	58	62	60	58
Al_2_O_3_	5	2	4	2	2	2	8
BaO	3	2	3	-	-	1	6
ZnO	8	9	8	8	6	6	8
MgO	12.5	13.5	12.5	16.5	14.5	14.5	12.5
Na_2_O	7.5	9.5	8.5	9.5	9.5	10.5	7.5
Yb_2_O_3_	6	6	6	6	6	6	-
*R*	1.103	1.000	1.069	1.000	0.871	0.933	1.000

Note: Related to [20], *R* = {[M_2_O] + [MO] + 3[M_2_O_3_]}/[P_2_O_5_], *R* = 1—metaphosphate glass, *R* < 1—ultraphosphate glass, *R* ˃ 1—pyrophosphate glass and [M*_x_*O*_y_*], the concentration of glass component in mol%.

## 4. Measurements of Glass Properties

The refractive index was measured at a temperature of 20 °C for a wavelength λ = 589.6 nm with a Abbe refractometer 4T (ATAGO, Tokyo, Japan). Three-side polished rectangular shaped glass samples with dimensions of 15 mm × 10 mm × 5 mm were prepared for the measurements. The measurement accuracy of this instrument is ±0.0002. An average value over six measurements for each glass sample was considered.

Transmittance and absorbance measurements of the glasses were performed with the spectrophotometer Cary 500 (VARIAN, Mulgrave, Australia) in the range of 200–3300 nm. Double-side polished samples with 2- and 5-mm thicknesses were used for the tests. The coefficient of thermal expansion (CTE) was measured with a horizontal quartz glass dilatometer Dil 801 (BÄHR Thermoanalyse GmbH, Hüllhorst, Germany) on specimens with dimensions of 30 mm in length and a 4 × 4-mm cross-section operating at 10 °C/min up to 10 °C above the dilatometric softening point (485–530 °C, according to the glass composition). CTE values were calculated in the 20–300 °C and 20–400 °C temperature ranges. The four characteristic glass temperatures related to the dynamic viscosity η = 10^14.6^; 10^13.4^; 10^13^ and 10^11^ P, were determined based on the obtained thermal expansion curve. In addition, the four characteristic glass temperatures related to lower viscosity η = 10^9^; 10^6^; 10^4^ and 10^2^ P were determined from the shape of the sample observation in a Leitz II A-P heat microscope (Esselte Leitz GmbH & Co KG, Stuttgart, Germany). A test cube with a 4-mm side length was used for the glass temperature measurements with an applied heating rate of 10 °C/min. The final viscosity curves (log_10_η(visc(P)) *vs.* temperature *T* (°C)) were determined based on the measurements in the dilatometer and in the heat microscope.

Preliminary information about the crystallization susceptibility of glass was obtained using the isothermal heating method (2 h at the temperature *T*_sph_, determined in a Leitz heat microscope that corresponds to glass viscosity η = 10^6^ P). The sample surface was observed after cooling down with an optical microscope to identify areas of excessive crystallization.

Differential scanning calorimetry (DSC) measurements were performed using a simultaneous thermal analyzer 449 F1 Jupiter (NETZSCH, Selb, Germany). The glass samples were heated up at a rate of 10 °C/min in an air atmosphere from 25 to 900 °C. The weight of the powder samples was 60 mg for each glass. Al_2_O_3_ was used as a reference material. The glass transition temperature *T*_g_ and the crystallization beginning temperature *T*_x_ were determined from the DCS curves using the onset method.

Diffractive X-ray measurements (XRD) were made using an X-ray powder diffractometer D500 (SIEMENS, Berlin, Germany) in the Bragg–Brentano geometry. The diffractometer was equipped with a high resolution semi-conductive Si:Li detector with a Cu lamp with K_α_ radiation source (wavelength λ = 1.5418 Å). Diffractograms were measured in stepping mode θ/2θ with step ∆_2__θ_ = 0.05^o^, a time of counting of 4 s/step and in the angle range of 2θ = 10°–60°. The thin layer samples in powdered form were placed on a monocrystalline chuck made of Si to eliminate the background from the base. Experimental data could be analyzed using the program XRAYAN for phase analysis, and the standard diffractogram ICDD (International Centre for Diffraction Data) PDF4 + 2009 base could be used for crystalline phase identification, if they appeared in analyzed samples.

The density of the glasses was measured at room temperature by the well-known Archimedes method of hydrostatic weighing. The measurements were executed using flat-polished parallel samples of glass with dimensions of 20 mm × 15 mm × 10 mm.

Vickers hardness (HV) tests were performed by a microhardness meter with micro-indenter (ZWICK, Einsingen/Ulm, Germany) under *Q* = 0.2 kG (1.96 N) loading. Hardness is defined as HV = 1.8544 × *Q*/*d*^2^, where d denotes the average diameter of the diamond prism indentation in glass.

The water durability of the glasses was qualified based on the glass sample mass decrease after 6 h of boiling in distilled water. The measurements were executed using mechanically flat-polished samples of glasses with dimensions of 20 mm × 15 mm × 10 mm. The sample mass decrease (Δm) after a 6 h-long exposure to boiling water, counted on 100 cm^2^ of sample surface (mg/100 cm^2^), is the measure of water durability of glass. The greater the value of decrease of sample mass, the lower the resistance of the glass against water interaction.

## 5. Development of Test Photonic Crystal Fiber

The IRF-16/02 glass, as a cladding material, and newly developed IRF-16/6Yb/F glass, as an active rod, were chosen for the test microstructured laser fiber fabrication. The difference of refractive indices was Δ*n* = 0.0009 for this two glasses, achieving depressed-index core in the final fiber, while measurement accuracy is ±0.0002. There are several methods for the development of fiber preform components (tubes and rods) with oxide glasses: extrusion; glass casting; and centrifugation [21,22,23]. We have developed tubes and rods using the casting method, followed by mechanical grinding and polishing.

The IRF-16/02 glass melt at 1120 °C is cast in a pre-heated (220 °C) graphite casting mold with a polished stainless steel cylinder in the center. After casting, the mold temperature rises up to 520 °C. The IRF-16/6Yb/F glass melt at 1220 °C is cast in an open rectangular graphite mold preheated to 475 °C. After the annealing process, run in an electric muffle furnace under nitrogen atmosphere, the casting mold is opened, and the glass is further mechanically processed. The core rod was cored out of a larger bulk sample with a diamond-embedded core drill, and the barrel of the rod was polished. The rod surface and both the outer and inner surfaces of the tubes are mechanically ground and polished to a high surface quality. Then, they are washed in ethanol and dried in a glove-box under a nitrogen atmosphere. Two kinds of raw tubes with 40/26 mm and 24.5/13 mm of external/inner diameters were prepared. The final tubes, micro-tubes and micro-rods are drawn with a fiber optic drawing tower under nitrogen protective atmosphere to eliminate glass contamination. The preforms for the sub-preform and final fiber drawing are made by the stack-and-draw technique. The manufactured fibers were not intended to have an outer polymer protective cladding, but they have good mechanical strength on bending and their critical radius is no more than 15 mm for a 285-μm diameter fiber. Several fiber samples with lengths of a few tens of meters were drawn giving a sufficient length for high power fiber laser construction (typically on the order of a few tens of centimeters is sufficient).

**Figure 7:**
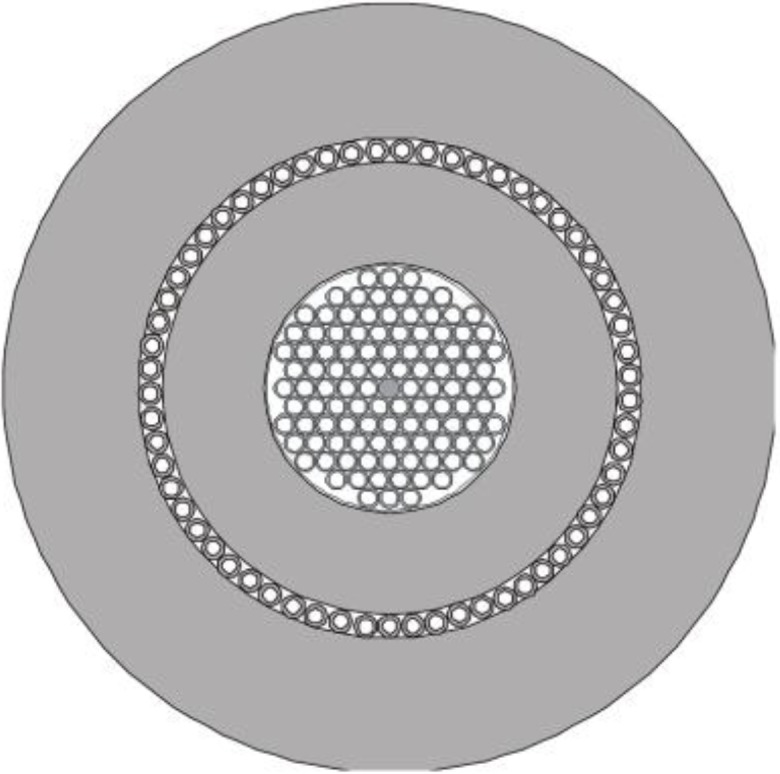
Design of the preform structure.

**Figure 8:**
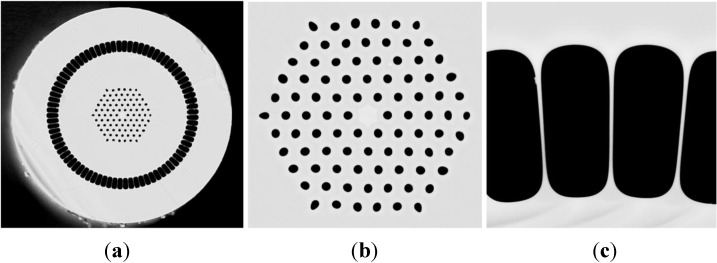
Cross-section of a manufactured double-clad photonic crystal fiber of the following dimensions: 285-μm outer diameter, 180-μm internal cladding (pump wave-guide) (**a**); 3.2-μm air holes, 11.5-μm core diameter, 8.5-μm diameter of the ytterbium-doped area (**b**); and 420-nm width of the glass bridges in air-cladding (**c**).

The double-clad structure with an air-cladding was designed and produced (Figure 7). The fiber was manufactured using the method of stacking capillaries and rods. The fiber was designed with an inner hexagonal lattice structure with 11 capillaries on the diagonal. One capillary was replaced by a doped rod resulting in a microstructure consisting of five rings of holes with a lattice constant of Λ = 7.5 µm, air holes *d* = 3.2 μm and relative hole size *d*/Λ = 0.43 (Figure 8). The manufactured fiber has a core of 11.5 μm in diameter. The doped area situated in the core has a diameter of 8.5 μm. The inner cladding has a diameter of 180 μm, which is suitable enough to couple with pumping diodes. The thickness of the outer air-cladding is about 15 μm, and the overall diameter of the fiber is 285 μm. The outer cladding in a double-clad structure was made from thin wall capillaries. The width of glass bridges in that area in the final fiber was measured to be 420 nm and the length was about 15 μm. The measured attenuation of the fiber was 6 dB/m for 1310 nm in single mode operation.

**Figure 9:**
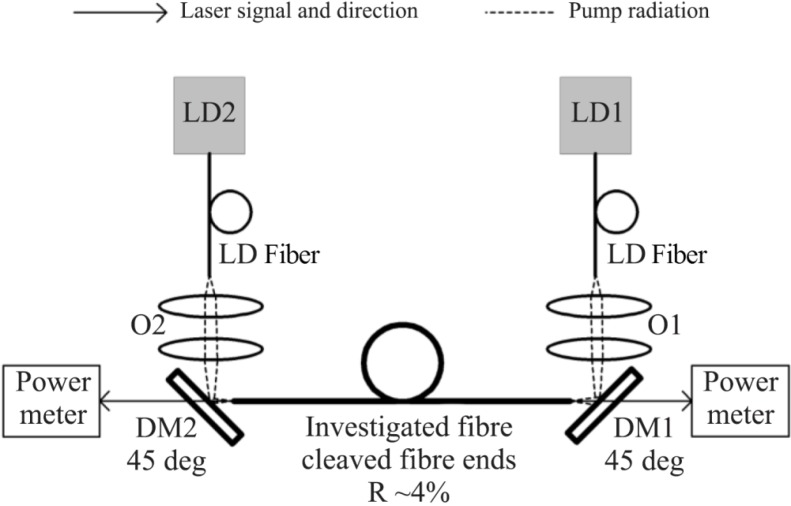
Experimental laser set-up: LD1, LD2, O1 and O2 indicate laser diodes and the radiation forming arrangement. DM1, DM2, dichroic mirrors; LD fiber, high power fiber delivering diode power.

**Figure 10:**
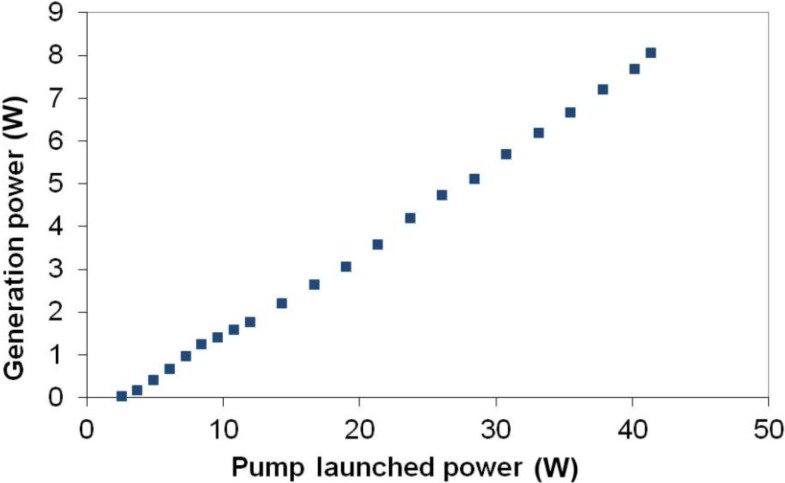
Output power *versus* launched power for experimental fiber laser.

**Figure 11:**
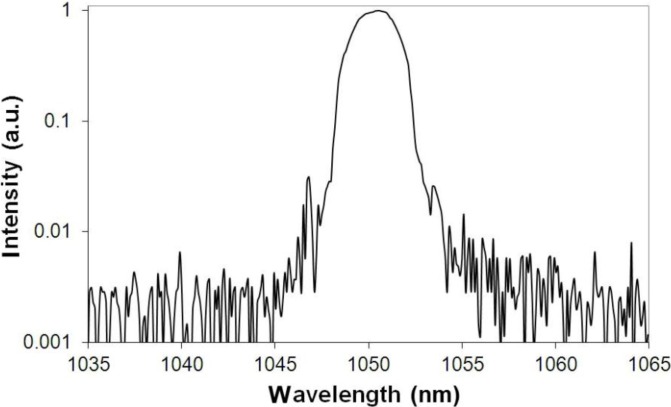
Spectrum of the laser emission at maximum output power.

Generation properties of the fibers were measured using laser set-up presented in Figure 9. The best performance was registered for a 53.6 cm-long fiber (Figure 10). The maximum power of 8.07 W generated from the 53.6 cm-long fiber was achieved. The laser operation wavelength was 1050.5 nm with a FWHM (Full Width at Half Maximum) of 4 nm. The slope efficiency was then 20.5%, taking into account the loss derived from dichroic mirror losses. The spectrum of the laser emission is presented in Figure 11. The resolution of the spectral measurement is 0.1 nm. A value of 1.59 and 1.44 of the *M_x_*^2^ and *M_y_*^2^ parameters was also measured in orthogonal directions for the generated laser mode using a beam profiler and measuring the diameter of the beam in the caustics. That relatively high value of the M^2^ parameter in the case of observed single-mode operation of our fiber is mainly caused by the hexagonal shape of the PCF lattice structure, which was also reported previously [24,25]. The value of the numerical aperture of the generated mode was as low as 0.07, which is characteristic for single-mode performance in photonic crystal fibers structures.

The IRF-16/02 and IRF-16/6Yb/F glasses were recently used for the successful fabrication of new types of LMA microstructured laser fibers with high power generation and excellent slope efficiency [26].

## 6. Conclusions

The thermally stable and highly ytterbium-doped (with a Yb_2_O_3_ content of 6 mol% (18 wt%; 15.7 × 10^20^ Yb^3+^/cm^3^) phosphate glass, carefully matched the refractive index and thermal properties (thermal expansion coefficient, viscosity characteristics) of the in-house synthesized meta-phosphate undoped glass synthesized in the P_2_O_5_-Al_2_O_3_-BaO-ZnO-MgO-Na_2_O oxide system, has been developed.

Both of the glasses were successfully used for the fabrication of a test dual-clad microstructured laser fiber with a regular predicted geometry and good laser properties. This confirmed the applicability of the developed glasses in the fabrication of active photonic crystal fibers.
